# Value of Periappendiceal Fat Sign on Ultrasound in Acute Appendicitis

**DOI:** 10.7759/cureus.16321

**Published:** 2021-07-11

**Authors:** Ayesha Walid, Azeemuddin Muhammad, Zainab Hussain

**Affiliations:** 1 Radiology, Dow University of Health Sciences, Civil Hospital Karachi, Karachi, PAK; 2 Radiology, Aga Khan University Hospital, Karachi, PAK

**Keywords:** ultrasound, acute appendicitis, periappendiceal fat echo, right lower quadrant pain, multiple detector computed tomography

## Abstract

Introduction

Acute right lower quadrant abdominal pain is one of the most common surgical presentations to the emergency department with acute appendicitis being the topmost differential diagnosis. Although computed tomography (CT) is the gold standard in diagnosing appendicitis, in our setup ultrasound is often the initial imaging modality available in urgent care settings especially for children and pregnant females. On ultrasound, an inflamed appendix has a diameter of 6 mm or more and is non-compressible. Increased periappendiceal fat echogenicity is an important ancillary sign of acute appendicitis that supports the sonographic diagnosis of acute appendicitis. To determine the association of periappendiceal fat echo sign (PFES) on ultrasound in surgically proven cases of acute appendicitis.

Methods

This cross-sectional study was held at the Department of Radiology at the Aga Khan University Hospital in Karachi, Pakistan. Periappendiceal fat echogenicity was assessed and prospectively graded in 59 patients. These patients had sonographic features of acute appendicitis which was later confirmed by surgery. Data were collected on a proforma and later analyzed. Frequency of increased periappendiceal fat echogenicity in acute appendicitis was calculated. Association of PFES with gender and ascites was evaluated with Fischer's exact test and with patient's age and appendiceal diameter was assessed using analysis of variance (ANOVA).

Results

Increased periappendiceal fat echogenicity was seen in 89.8% of patients with acute appendicitis. 10.2% of patients had acute appendicitis with normal surrounding fat. Mean appendiceal diameter in patients with grade 3 PFES was significantly more than those with grade 2 or grade 1 PFES. PFES had no association with age and gender of the patient or with ascites.

Conclusion

Increased periappendiceal fat echogenicity is an important ancillary sign of acute appendicitis that helps support its sonographic diagnosis.

## Introduction

Acute appendicitis is the most common surgical emergency affecting individuals of all ages [1]. The life-time risk of developing acute appendicitis has been reported as 9% for males and 7% for females [2].

Patients presenting with characteristic clinical signs and symptoms of acute appendicitis go through instant surgery without radiological workup. In patients with atypical or confusing clinical findings radiological workup is requested. The selection of modality whether US or CT in this clinical scenario is mainly reliant on institutional preference and availability of skilled experts, although patient demographics and BMI are important influencing factors [3].

The stated sensitivities for abdominal CT are 90%-100%, specificities ranging from 91%-99%, 94%-98% accuracy, positive predictive value (PPV) and negative predictive value (NPV) of 92%-98% and 95%-100%, respectively, for the identification of acute appendicitis [3-6]. The most important imaging study in the evaluation of patients with atypical presentations of appendicitis is abdominal CT. In selected patients with suspected appendicitis, studies have reported a decrease in negative laparotomy rate and appendiceal perforation rate when pelvic CT was used [7-9].

The two most predictive signs, that is, the signs with the highest probability of a correct diagnosis are peri-appendiceal fat stranding and appendiceal diameter [10]. No other sign is needed to increase the level of confidence for diagnosing appendicitis. In a prospective study, comparing US and CT, Balthazar et al. indicated CT to be superior to graded compression US in the diagnosis of acute appendicitis with similar specificities (89% vs. 91%, respectively) and PPV (96% vs. 95%, respectively) [11].

Despite the superior diagnostic parameters of CT scan, US is often the first line of investigation as it is simple, rapid, easily available, inexpensive and not associated with ionizing radiation [1-4]. Due to the lack of non-ionising radiation and dynamic ability ultrasound is the imaging modality of choice in the evaluation of suspected acute appendicitis and allows the radiologist to clinically assess the patient. It is the initial imaging examination of choice, particularly in women of childbearing age and children [12]. As sonography involves a short acquisition time, not using ionizing radiation, and other causes of abdominal pain such as ovarian cysts, tubo-ovarian abscesses and mesenteric adenitis may be diagnosed, it can be performed at the bedside [2,10]. The course of the appendix is variable hence making the visualization of the structure slightly challenging including both retrocecal and pelvic locations. A retrocecal appendix can be best visualised on scans acquired with the transducer position next to the caecum or to the ascending colon, with an oblique plane of insonation [13]. A target appearance, characterized by a fluid-filled center and surrounded by an echogenic mucosa and submucosa and hypoechoic muscularis, is visualised when imaging in the axial plane [14]. Endovaginal scanning is the best method for visualization of the pelvic appendix in women. The ability to see a pelvic appendix will be influenced by different degrees of bladder filling [15].

The ultrasound criteria for acute appendicitis include visualization of a non-peristaltic, non-compressible, tubular, blind-ending structure with a diameter of 6 mm or more in the right iliac fossa [13,16]. However, there are many ancillary signs of acute appendicitis that can help in its sonographic diagnosis. These include prominent hyperechoic mesoappendix or pericecal fat, localized periappendiceal fluid collection, aperistaltic bowel loops, enlarged lymph nodes and presence of free fluid [1,6].

US with graded compression has a sensitivity of 89% and specificity of 100% and is a widely used technique within the diagnosis of acute appendicitis [5]. In graded compression technique, pressure is applied through a high-resolution linear transducer for displacement and compression of the underlying bowel loops, thereby making the appendix visible. The visualization of the psoas muscle and iliac vessels should be part of a proficient exam.

The objective of this study is to evaluate whether periappendiceal fat echo sign (PFES) is an important ancillary sign that would be helpful for the sonographic diagnosis of acute appendicitis and to determine its frequency in surgically proven cases of acute appendicitis. 

## Materials and methods

The study was carried out at the Aga Khan University hospital in Karachi, Pakistan. The study design was prospective and cross-sectional. This was a residents dissertation and the research training and medical center of the College of physicians and surgeons Pakistan had supervised the research ensuring that all ethical guidelines were met against this study. The sample size estimation was done on the World Health Organization software determinations of sample size. The prevalence of increased intrabdominal fat echo has been reported to be 89% [17]. Thus at a confidence level of 95% and significance estimated at a p-value of 0.05, 59 patients were enrolled for the study with margin of error calculated within 6%. Non-probability purposive sampling technique was deployed for recruiting patients

The study enrolled all consecutive patients of all ages and both genders who were referred to our department for US examination with clinical suspicion of acute appendicitis (periumbilical/right lower abdominal pain, vomiting and fever) followed by surgery and histopathology at the same institution. Patients in whom adequate ultrasound examination could not be performed or the appendix was not visualized (retrocecal location) were excluded as well as those patients who were treated conservatively or did not have surgical findings of appendicitis.

**Figure 1:**
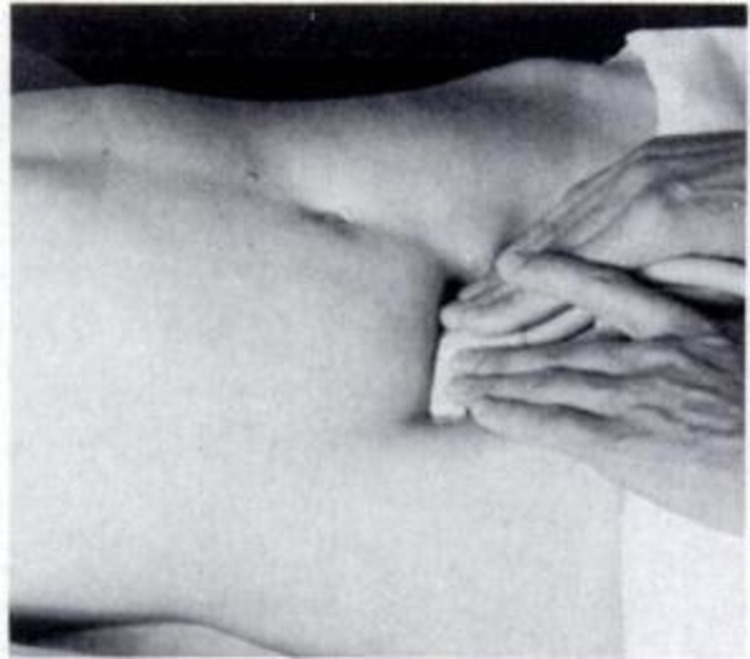
Ultrasound graded compression technique by Puylaert. Puylaert JB. Acute appendicitis: US evaluation using graded compression. Radiology. 1986 Feb;158(2):355-60.

Informed consent was obtained by the principal investigator. US was performed on Toshiba Nemio using a high frequency (4-8 MHz ) linear array and convex (3-5 MHz ) array transducers. Graded compression technique described by Puylaert et al. was followed for US examination [18]. Uniform pressure was applied over the right iliac fossa via the handheld ultrasound transducer. Resultantly normal bowel loops were not visualized due to flattening between the layers of the abdominal wall musculature or displacement from the image (Figure 1).

Ultrasound examination for acute appendicitis was performed by a consultant radiologist with five-year clinical experience. Any abnormal loop of gut or the obstructed appendix was non-compressible and adequately visualized on the graded compression image. An enlarged appendix with an outer diameter equal to or greater than 6 mm showing lack of compressibility is defined as acute appendicitis. The periapendiceal fat echo sign in the right iliac fossa was assessed and classified on the scale developed by Lee et al. [7]. Grade 1 was described as normal-appearing fat which is hypoechoic to the adjacent appendix with grade 2 showing increased echogenicity of the periappendiceal fat; however, underlying muscles and vessels still visualized and grade 3 with the periappendiceal fat appearing hyperechoic with obscuration of the underlying muscles and vessels

Comparison was made with the contralateral abdominal fat echo during the sonographic examination. Surgical and histopathology findings regarding final diagnosis were obtained from medical records of patients by the principal investigator. All findings were recorded on standard Performa.

Statistical analysis was performed with Statistical Package for the Social Sciences (SPSS) for Windows, Version 16 (SPSS Inc., Chicago, IL). Descriptive analysis was conducted with frequencies and percentages for categorical variables such as gender while mean and standard deviation were estimated for continuous variables like age and appendicular diameter. The presence of the PFES on ultrasound was assessed for surgically proven cases of acute appendicitis. Association of PFES with categorical variables like sex and ascites was further assessed with Fischer's exact test. Two-sided ANOVA was applied to see the association of PFES with continuous variables like age and appendicular diameter. A p-value of < 0.05 was considered statistically significant.

## Results

A total of 59 patients were included in this study. All these patients were diagnosed with acute appendicitis on ultrasound and were followed up with surgery and histopathology at our institution which was positive for appendicitis. There were 36 (61%) male and 23 (39%) female patients. Age range was seen between 5 and 62 years with mean age of 19.5 years with standard deviation of 12.3. PFES was assessed and graded into three categories in all patients. Six out of 59 patients (10.2%) showed normal periappendiceal fat echogenicity, corresponding to grade 1 PFES. Thirty-four patients (57.6%) showed grade 2 PFES. Nineteen patients (32.2%) had grade 3 PFES. Thus increased periappendiceal fat echogenicity was seen in a total of 53/59 patients (89.8%). Presence and absence of ascites with acute appendicitis was also taken into account. Ascites was seen in 25/59 patients (42.4%). Thirty-four patients (57.6%) had no ascites as seen in Table 1.

**Table 1:** Baseline data. PFES: periappendiceal fat echo sign; N: total patients; SD: standard deviation.

Study variables	N = 59	(%, ±SD)
Gender		
Male	36	61%
Female	23	39%
Age (5-62 years)	19.5	±12.3
PFES		
Grade 1	6	10.2%
Grade 2	34	57.6%
Grade 3	19	32.2%
Ascites		
Present	25	42.4%
Absent	34	57.6%

Association between appendiceal diameter in acute appendicitis and PFES was assessed with ANOVA. Our results showed that the mean appendiceal diameter in patients with grade 3 PFES was 1.1 cm. This was significantly more than patients with normal periappendiceal fat echo and those with increased periappendiceal fat echo with visualization of underlying structures with a p-value of 0.002 which rendered it significant.

Mean appendiceal diameter was not significantly different in patients with grade 1 PFES (0.82 cm) and those with grade 2 PFES (0.9 cm) as seen in Table 2.

**Table 2:** Association between appendiceal diameter and periappendiceal fat echo sign. PFES: periappendiceal fat echo sign.

PFES	Appendix diameter	p-value
Grade 1	0.82 cm	>0.05
Grade 2	0.90 cm	>0.05
Grade 3	1.1 cm	0.02

Association of PFES with gender and ascites was evaluated with Fischer exact test. A p-value of 2.7 and 2.5 was obtained, respectively, indicating no significant association of gender and the presence of ascites in the sample cohort.

Two-way ANOVA was applied to see how PFES varied with the age of the patient. Mean age of patients with grade 3 PFES (23 years) was more than those with grade 2 (19 years) and grade 1 PFES (12 years). This difference was, however, not statistically significant (p = 0.16).

## Discussion

Acute appendicitis is one of the commonest causes of acute abdomen presenting to the emergency department that often warrants immediate surgical intervention. In most of the cases, the diagnosis is straight forward based on typical clinical and laboratory findings. However, 20%-30% of patients may present with atypical/subtle clinical picture [19]. Also there is a vast list of differential diagnosis in patients presenting with acute RLQ pain. Imaging is therefore crucial to establish or negate the diagnosis of acute appendicitis and to rule out other causes of right-lower quadrant (RLQ) pain.

The most specific sign of acute appendicitis on US is a dilated non compressible appendix with a diameter more than 6 mm [16]. There are also a few ancillary signs of acute appendicitis that further support its sonographic diagnosis.

In a study by Lee et al. 89% of patients with acute appendicitis revealed inflammatory changes on ultrasound examination [7]. They assessed the importance of increased intra-abdominal fat echo in the sonographic evaluation of acute right lower quadrant pain. The echogenicity of the intra-abdominal fat was graded in three categories :

1. Normal,

2. Slight increase with visualization of the underlying muscles and vessels, and

3. Marked and diffuse increase with obscuration of the underlying structures.

They reported the diagnostic accuracy as 81% with a sensitivity of 73%, specificity of 98%, and positive and negative predictive value of 9% and 64% respectively for increased intraabdominal fat echo for surgically proven appendicitis [7].

Intra-abdominal fat is packed between the abdominal viscera and consists of the greater omentum and small bowel mesentery in the RLQ. It serves both as a pathway and a barrier for disease spread. Inflammatory conditions as acute appendicitis will therefore be associated with infiltration of the adjacent fat leading to an increase in its echogenicity [7]. These findings were also reported by Noguchi et al. who reported that progressive inflammation is signified by the presence of a hyperechoic periappendiceal surrounding fat possibly representing omental or mesenteric spread of inflammation around the appendix [3].

In some clinical situations when the appendix is not directly visualized on US, as retrocecal position or appendiceal perforation, increased RLQ fat echogenicity can also serve as a warning sign, necessitating further imaging.

Our study showed that PFES is an important ancillary sign that helps in the sonographic diagnosis of acute appendicitis. Periappendiceal fat echogenicity was increased in 53/59 (89.8%) of our patients which is similar to the results of the western literature [7,20].

In a study by Lee et al. diagnostic utility of increased intra-abdominal fat echo during the sonographic evaluation of 328 patients with acute RLQ pain was assessed [7]. Multiple etiologies (inflammatory) such as acute appendicitis, right colonic diverticulitis, pelvic inflammatory disease, enteritis and others were evaluated and a definite diagnosis was established with surgical/pathological findings or clinical follow-up. They observed that increased intra-abdominal fat echogenicity was seen more commonly in patients with a positive final diagnosis (73%) as compared to patients with a negative one. They calculated the sensitivity, specificity, accuracy, PPV and NPV of increased intra-abdominal fat echogenicity for a positive histopathology outcome as 73%, 98%, 81%, 99% and 64% respectively. They also concluded that increased intra-abdominal fat echo was seen in 100% cases of right colonic diverticulitis and 89% of acute appendicitis. In this study, we assessed the frequency of increased periappendiceal fat echogenicity in patients with sonographic diagnosis of acute appendicitis which was later confirmed with surgery/histopathology. Our results of 89.8% are comparable with those of Lee et al.

Another study conducted by Kessler et al. prospectively evaluated 125 patients for appendiceal and periappendiceal US features of appendicitis. They concluded that a diameter of 6 mm or larger, was the most accurate radiological finding for appendicitis having a sensitivity, specificity, NPV, and PPV of 98%. Inflammatory fat changes were present in 91% of patients and were the most accurate finding of appendicitis [20]. This is also similar to our result of 89.8%.

In a retrospective study by Noguchi et al. also concluded that the presence of a periappendicular hyperechoic structure is suggestive of advanced appendicular inflammation and its accompanying complications [3]. They studied a small set of 25 patients and correlated the prevalence of periappendicular hyperechoic structure with the pathologic severity of appendicitis.

Their results showed that periappendiceal hyperechoic structure was seen with 100%, 29% and 0% cases of gangrenous, phlegmonous and early appendicitis respectively. They also went on to conclude that the incidence rates of perforation, infected exudate or abscess formation and prominent adhesions to the surrounding tissue was higher in patients with positive periappendiceal hyperechoic structure.

**Figure 2:**
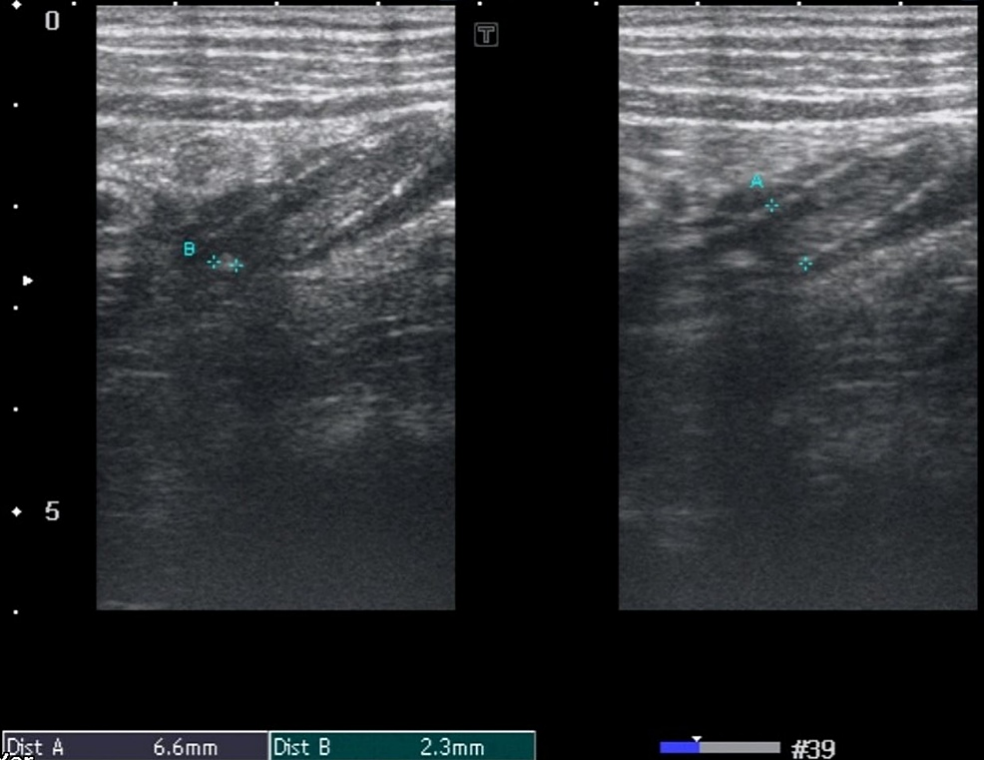
Grade 1 PFES. High-frequency probe demonstrates acute appendicitis measuring 6.6 millimeters (A) with appendicolith measuring 2.3 millimeters (B) showing grade 1 PFES (periappendiceal fat echo sign).

**Figure 3:**
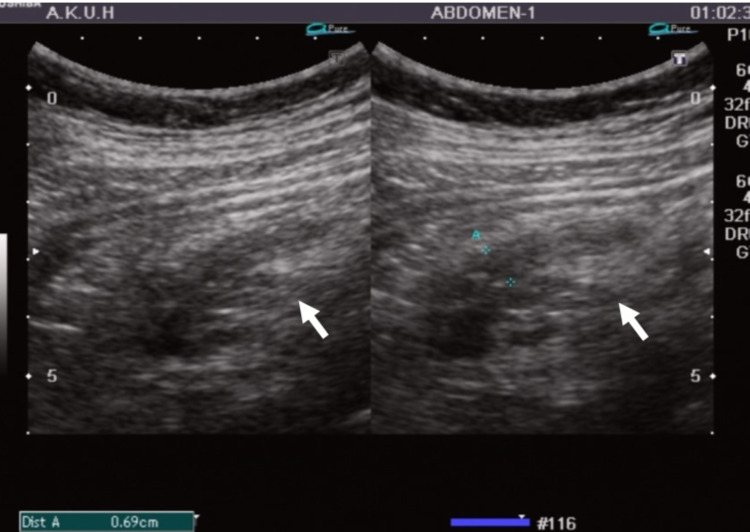
Grade 2 PFES. 13 years with history of lower abdominal pain. Ultrasound of the right iliac fossa demonstrates acute appendicitis with transverse dimension of 0.69 centimeters (A). Periappendiceal fat echo sign (PFES) is seen (white arrow); however, the underlying vessels and muscles can be visualized.

**Figure 4:**
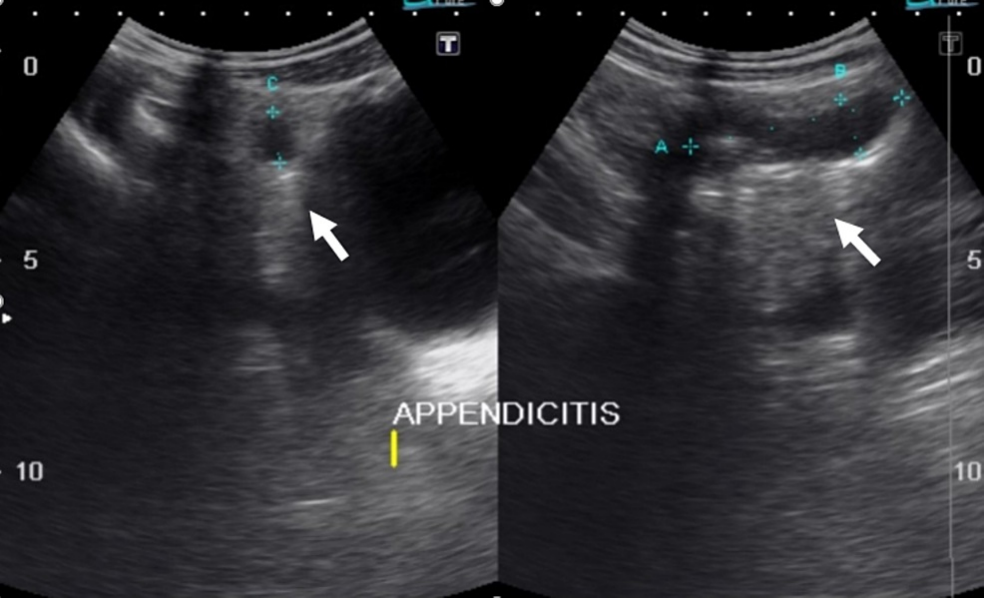
Grade 3 PFES. 13 years male presented with lower abdominal pain and vomiting. Ultrasound of the right iliac fossa reveals a dilated blind-ending bowel loop in RIF measuring 5 centimeters in length (A) and 1.2 centimeters in short axis (B) representing acute appendicitis. An appendicolith also noted within the appendicular lumen. Periappendiceal fat echo (white arrow) is so echogenic that the underlying structures are not visualized consistently with grade 3 PFES (periappendiceal fat echo sign).

In our study, we have evaluated the effect of appendiceal diameter on PFES. Our results show that the mean appendiceal diameter in patients with grade 3 PFES (1.1 cm) is significantly more than those with grade 1 (0.82 cm) and grade 2 (0.9 cm) PFES. This supports the fact that with severe inflammation in which we see increased appendiceal diameters there is significant surrounding fat inflammation and the periappendiceal fat echogenicity is so much increased that even the underlying normal structures cannot be visualized on ultrasound (Figures 2, 3, 4).

The effect of other confounding variables on PFES was also evaluated in our study. Our results show that patient's age, sex and ascites have no significant association with PFES in acute appendicitis. No such association has been described in the local and the international literature as well [21,22].

There were six patients in our study group who met the sonographic criteria of acute appendicitis, however, showed normal echogenicity of periappendiceal fat. On histopathology no evidence of inflammatory changes was identified in the surrounding mesentery. This signifies that periappendiceal fat echogenicity is not increased in all patients of acute appendicitis as these patients likely present with early/mild inflammation and have a deep-seated appendix such as retrocecal location or inflammation confined to a segment such as tip appendicitis. Inflammatory fat changes are also difficult to assess in slim patients due to paucity of intra-abdominal fat. This finding was also reported in previous studies [7,20].

In our study, we had a relatively high proportion of male patients. Age range was between 5 and 62 years with mean age of 19.5 years for acute appendicitis. This is in keeping with the local literature which also provides similar statistics for acute appendicitis [23,24].

There were a few limitations to our study. First, as it was a single-center study. Second, a single radiologist performed all the US examinations. Considering the subjective nature of the US examination we could have employed two or more radiologists and calculated their interobserver variability however as this was not one of the objectives it does not hamper the study results. Third, only those patients were enrolled in the study in whom the appendix was directly visualized. Those patients in whom there was increased RLQ fat echogenicity with clinical suspicion of acute appendicitis; however, appendix was not visualized were not further followed.

## Conclusions

Increased periappendiceal fat echogenicity is an important indicator of appendiceal inflammation. It supports the sonographic finding of acute appendicitis in such patients in whom the inflamed appendix is directly visualized. In cases where the appendix is not visualized on sonographic examination increased RLQ fat echogenicity should prompt further investigation with CT scan.
